# Two-dimensional magnetotransport in a black phosphorus naked quantum well

**DOI:** 10.1038/ncomms8702

**Published:** 2015-07-07

**Authors:** V. Tayari, N. Hemsworth, I. Fakih, A. Favron, E. Gaufrès, G. Gervais, R. Martel, T. Szkopek

**Affiliations:** 1 Department of Electrical and Computer Engineering, McGill University, 3480 rue Université, Montréal, Québec H3A 2A7, Canada.; 2 Department of Physics, Université de Montréal, 2900 boul. Édouard-Montpetit, Montréal, Québec H3C 3J7, Canada.; 3 Department of Chemistry, Université de Montréal, Montréal, 2900 boul. Édouard-Montpetit, Québec H3C 3J7, Canada.; 4 Department of Physics, McGill University, 3600 rue Université, Montréal, Québec H3A 2T8, Canada.; 5 Department of Chemistry, Université de Montréal, 2900 boul. Édouard-Montpetit, Montréal, Québec H3C 3J7, Canada.

## Abstract

Black phosphorus (bP) is the second known elemental allotrope with a layered crystal structure that can be mechanically exfoliated to atomic layer thickness. Unlike metallic graphite and semi-metallic graphene, bP is a semiconductor in both bulk and few-layer form. Here we fabricate bP-naked quantum wells in a back-gated field effect transistor geometry with bP thicknesses ranging from 6±1 nm to 47±1 nm. Using a polymer encapsulant, we suppress bP oxidation and observe field effect mobilities up to 900 cm^2^ V^−1^ s^−1^ and on/off current ratios exceeding 10^5^. Shubnikov-de Haas oscillations observed in magnetic fields up to 35 T reveal a 2D hole gas with Schrödinger fermion character in a surface accumulation layer. Our work demonstrates that 2D electronic structure and 2D atomic structure are independent. 2D carrier confinement can be achieved without approaching atomic layer thickness, advantageous for materials that become increasingly reactive in the few-layer limit such as bP.

## Abstract (web summary)

The two-dimensional atomic layers of black phosphorus may be exfoliated to create devices with desirable electronic transport properties. Here, the authors observe two-dimensional quantum transport in black phosphorus quantum wells, protected from oxidation by encapsulation in a polymer layer.

## Graphical abstract

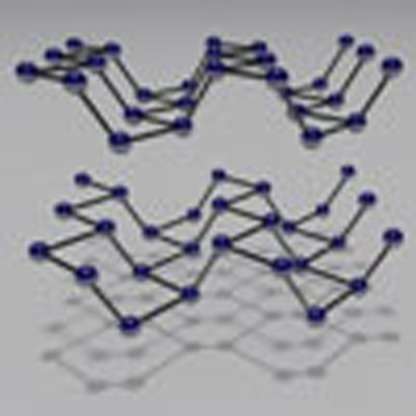

Layered two-dimensional (2D) materials have undergone a renaissance since the development of mechanical exfoliation techniques^1^. Black phosphorus (bP) is a layered material (Fig. 1a) with van der Waals interlayer bonding^2^, and is the only elemental allotrope other than graphene that is presently known to be a 2D material. Recent work has shown that bP can be exfoliated down to the atomic limit^3^,^4^,^5^,^6^,^7^,^8^. In bulk form, bP is a narrow gap semiconductor with a 0.3 eV direct bandgap^9^, which grows to an ∼2 eV bandgap in the atomic monolayer limit^2^, ideal for application to transistors^10^. Ambipolar conduction, mobilities approaching ∼1,000 cm^2^ V^−1^ s^−1^ and anisotropic conductivity have been demonstrated^3^,^4^,^5^, leading to a revitalized interest in bP^11^.

Interestingly, it was long ago observed^2^ that despite the weak van der Waals bonding between the 2D atomic layers of bP, the effective mass for electron (hole) motion between planes is remarkably light at 0.13*m*_0_ (0.28*m*_0_)^12^, where *m*_0_ is the free-electron mass. Exfoliated bP layers are thus effectively naked quantum wells with a low charge trap density at the bP surface due to the absence of broken covalent bonds and the simultaneous delocalization of charge carriers across atomic layers due to the light effective mass. The high electronic quality of the naked bP surface, requiring no passivation, is rare among semiconductors.

In our work, we fabricate field effect transistors (FETs) with exfoliated bP layers ranging in thickness from 6±1 nm to 47±1 nm (11±2 to 90±2 atomic layers). Despite being the most stable allotrope of phosphorus, bP suffers from photo-oxidation in a reaction that proceeds faster as atomic film thickness is approached^6^. The deleterious effects of photo-oxidation are mitigated by using bP layers thicker than a few atomic layers, by encapsulating the bP in a polymer superstrate, and by minimizing exposure to oxygen, water and visible light. We measure the electronic transport properties of bP FETs over the temperature range of 0.3 to 300 K, including the measurement of Shubnikov-de Haas (SdH) oscillations at magnetic fields up to 35 T. The observed SdH oscillations indicate the presence of a 2D hole gas in an accumulation layer as in conventional semiconductor heterostructures, demonstrating that 2D carrier confinement can be achieved in bP of ∼90 atomic layer thickness, which are much less susceptible to photo-oxidation than few-layer bP. Independent studies of bP on exfoliated hexagonal boron nitride (hBN)^13^, and bP encapsulated within exfoliated hBN layers^14^,^15^ report very similar observations of 2D magnetotransport.

## Results

### Device structure and characterization

**Figure 1:**
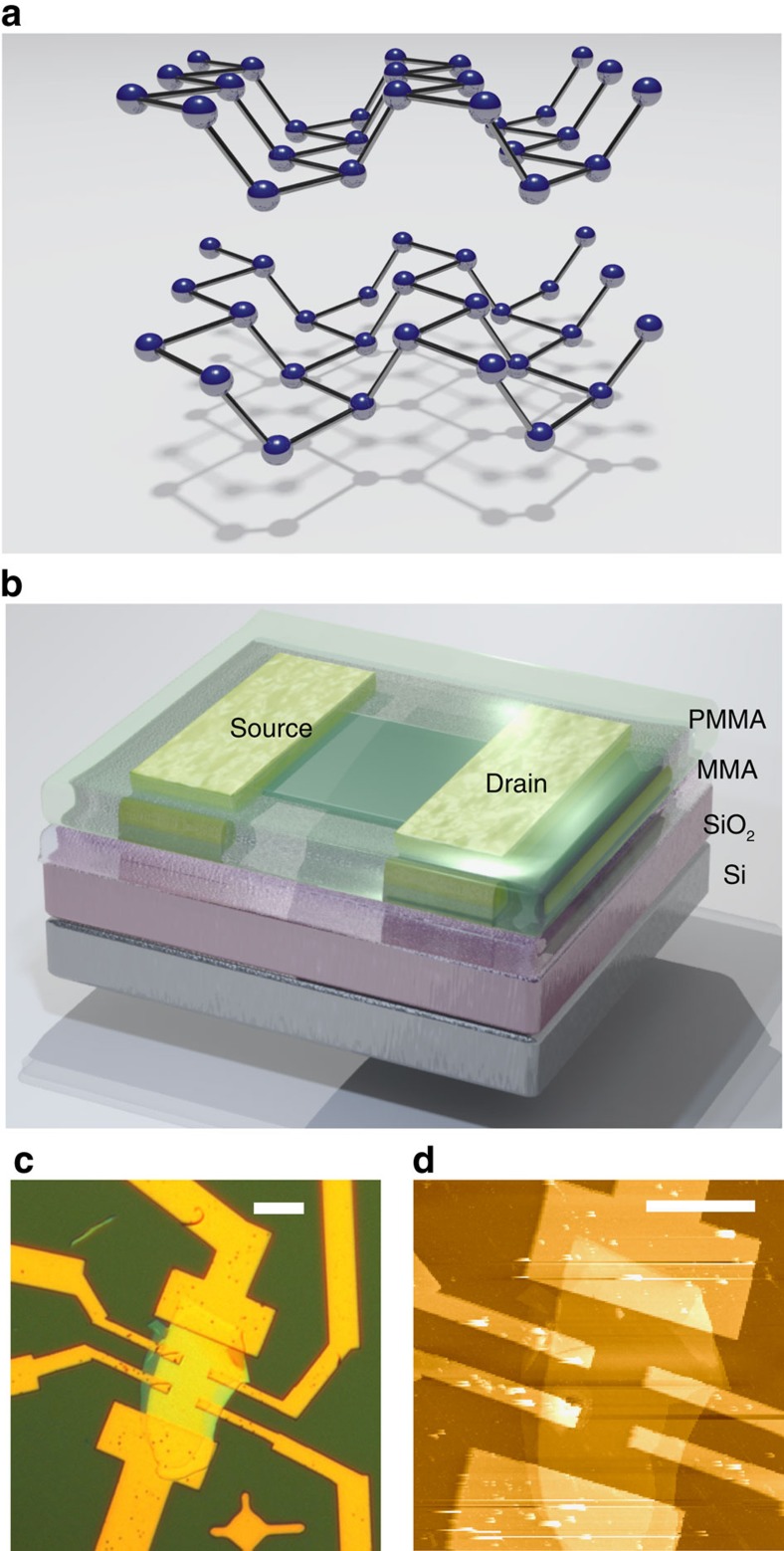
Structure of black phosphorus FETs. (**a**) The bP crystal structure is composed of puckered honeycomb layers with an interlayer distance of 5.24 Å. (**b**) Three-dimensional schematic view of a bP FET with oxidized silicon back-gate and an encapsulating layer of MMA and PMMA. (**c**) Optical image of an encapsulated bP FET in Hall bar geometry. Scale bar, 10 μm. (**d**) AFM image of the same device with encapsulating layer removed. The bP thickness is 43±2 nm (82±4 atomic layers). Scale bar, 10 μm.

Ultra-thin bP samples were prepared by mechanical exfoliation from bulk bP crystals using a polydimethylsiloxane (PDMS) stamp technique^6^. The sample substrates were degenerately doped Si wafers, with 300 nm of dry thermal oxide to allow rapid optical identification of bP flakes and back-gating over a wide temperature range. To protect bP FETs against degradation, 300 nm of copolymer (methyl methacrylate) and 200 nm of polymer (polymethyl methacrylate) were deposited. A schematic of the bP FET structure is shown in Fig. 1b. The polymer layer forms a water-impermeable superstrate-suppressing oxidation. An optical reflection image under white light illumination of a typical encapsulated bP FET is shown in Fig. 1c. Upon completion of electron transport measurements, described in detail below, Raman spectroscopy was performed (see Methods). The encapsulating polymer was then removed with acetone and the bP layer thickness was measured by atomic force microscopy (AFM) within a glove box. An AFM image of an unencapsulated bP FET is shown in Fig. 1d. The bP layer thickness of this representative device in Hall bar geometry was determined to be 43±2 nm (82±4 atomic layers). Importantly, the bP surface is free of the surface roughening that arises from oxidation^6^,^7^,^8^,^16^, despite exposure to ambient conditions. Encapsulation with a PMMA/MMA superstrate, similar to encapsulation with parylene^6^ or AlO_*x*_ (ref. 16), was thus found to be an effective means to suppress photo-oxidation of multi-layer bP. Most recently, hBN has also been used to preserve bP quality^14^,^15^.

### Zero-field electron transport

**Figure 2:**
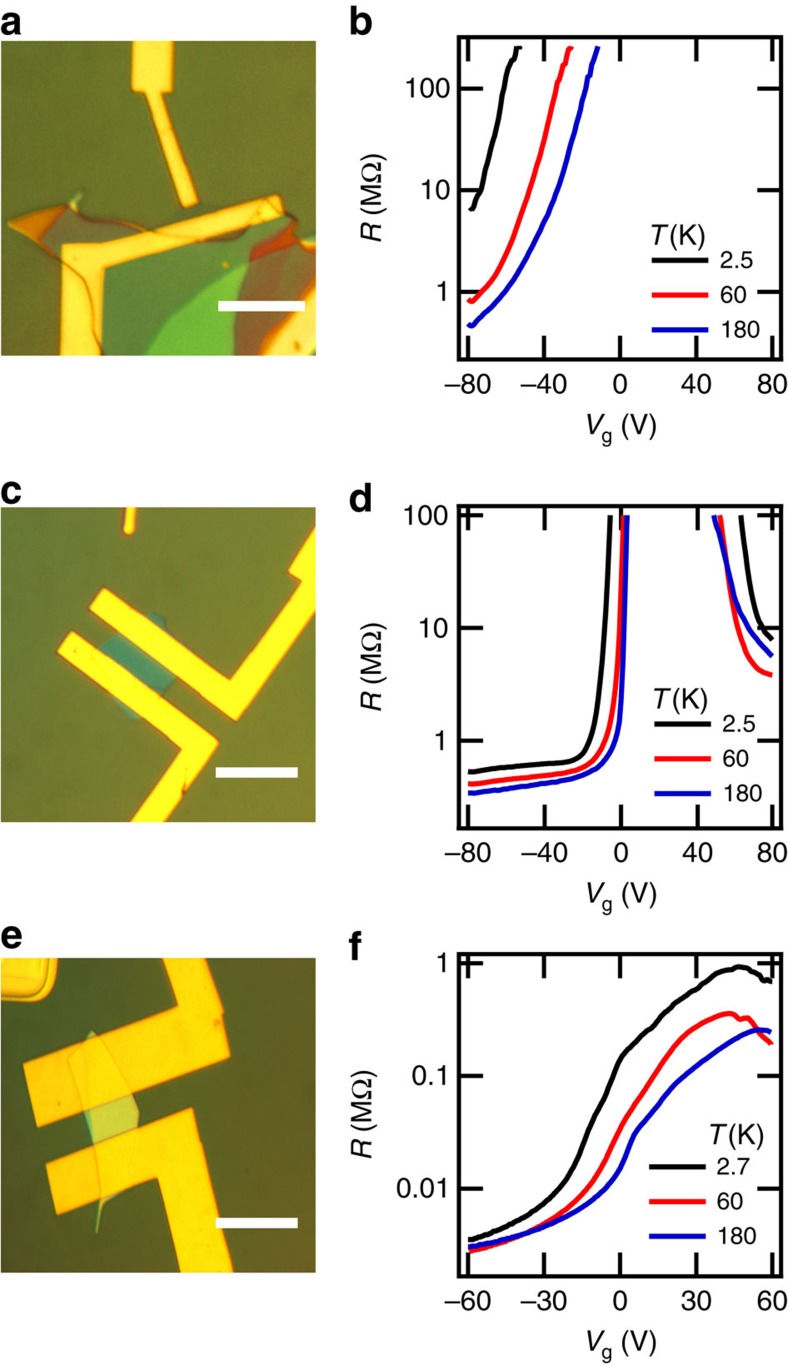
Black phosphorus FET characterization. (**a**,**c**,**e**) Optical images of encapsulated devices with increasing thickness of 6±1 nm, 12.5±1 nm and 47±1 nm, respectively. Scale bar, 5 μm. (**b**,**d**,**f**) Source-drain resistance *R* as a function of gate voltage *V*_g_ measured with an ac source-drain bias at different temperatures for the devices shown in **a**,**c**,**e**, respectively. The thickest 47±1 nm device exhibits a resistance two orders of magnitude smaller than the devices of thickness 12.5±1 nm and 6±1 nm.

Charge transport was investigated in over 40 bP FETs, of which three representative samples are shown in Fig. 2. The thinnest bP FET measured was 6±1 nm (11±2 atomic layers) thick, displayed in Fig. 2a. The source-drain resistance *R* measured by ac lock-in technique at ∼10 Hz versus gate voltage *V*_g_ with a source drain bias of *V*_sd_=10 mV is illustrated in Fig. 2b at different temperatures. The resistance exceeded our measurement limit for electrons, while strong insulating behaviour, ∂*R*/∂*T* < 0, was observed for holes. A sample of 12.5±1 nm (24±2 atomic layers) thickness is displayed in Fig. 2c, with measured resistance in Fig. 2d. Ambipolar conduction is observed, with a threshold for hole conduction at a gate voltage *V*_g_∼−15 V and a threshold for electron conduction at *V*_g_∼65 V at low temperature. The bP FET channel is thus slightly hole doped. A weaker temperature dependence of the resistance *R* at high hole density is observed as compared with the thinner 12.5±1 nm device. The thickest bP FET measured was 47±1 nm (90±2 atomic layers) thick, displayed in Fig. 2e. The measured resistance *R*, plotted in Fig. 2f, is two orders of magnitude smaller than that in the aforementioned devices. The thickest device displays a low on/off current ratio of 10^2^ at 180 K. In contrast, on/off current ratios exceeding 10^5^ can be achieved in thin samples. The field effect hole mobility, *μ*_FE_ = (*L*/*W*) × ∂(1/*R*)/∂(*CV*_g_) where *C* is the gate capacitance per unit area for a channel of length *L* and width *W* reaches ≈600 cm^2^ V^−1^ s^−1^ at *V*_g_ ≈−20 V for the 47±1 nm device at temperatures *T* <80 K. The field effect mobility of our 43±2 nm thick bP Hall bar reaches ≈900 cm^2^ V^−1^ s^−1^ at 300 mK and is the highest field effect mobility observed in our experiments. The electrodes in our samples are not aligned with the crystallographic axes of the bP layers, and thus our observed mobility is an average over the anisotropic transport properties of bP^4^. Further details concerning temperature-dependent field effect mobility measurement are described in the Methods section.

### High-field magnetotransport

**Figure 3:**
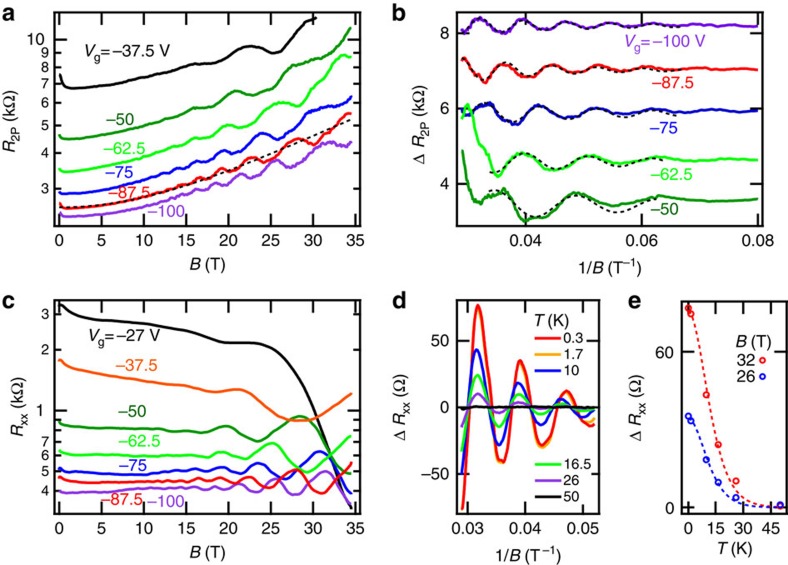
Shubnikov-de Haas oscillations. (**a**) The measured resistance *R*_2P_ of a 47±1 nm two-terminal bP FET on a log-scale as a function of magnetic field *B* applied normal to the bP layer at different gate voltages at *T*=0.3 K. A weak localization peak at low field, a slowly varying positive magnetoresistance, and SdH oscillations are observed. The slowly varying positive magnetoresistance has a parabolic form, with an example of a best-fit shown by the dashed line. (**b**) Subtracting the magnetoresistance background, the oscillating resistance Δ*R*_2P_ is plotted as a function of 1/*B*, with vertical offsets for clarity. The SdH oscillations were fit to the Lifshitz-Kosevitch formula, indicated by dashed lines. (**c**) The measured longitudinal resistance *R*_XX_ of a 47±1 nm Hall bar bP FET on a log-scale as a function of magnetic field *B* applied normal to the bP layer at different gate voltages at *T*=0.3 K. SdH oscillations are observed. (**d**) The oscillatory longitudinal resistance Δ*R*_XX_, determined by subtraction of a parabolic best-fit to the slowly varying background resistance, plotted versus 1/*B* at constant gate voltage *V*_g_=−100 V and varying temperature *T*. The SdH oscillation amplitude decreases with increasing temperature. (**e**) The longitudinal resistance Δ*R*_XX_ maxima at *B*=26 T and *B*=32 T at gate voltage *V*_g_=−100 V plotted versus temperature *T* is indicated with circles. Dashed lines show a best-fit to the thermal damping function *R*_0_ × Λ/sinh(Λ).

To further elucidate the nature of hole conduction in bP, the magnetoresistance of our 47±1 nm thick two-terminal bP FET and 43±2 nm thick Hall bar bP FET were measured at a fixed low temperature, *T*=0.3 K in a magnetic field up to *B*=35 T. The two-point source-drain resistance *R*_2P_ of the 47±1 nm thick bP FET was measured by an ac lock-in technique with a 10 nA bias current, and is plotted in Fig. 3a versus magnetic field oriented normal to the bP atomic planes at different gate voltages *V*_g_. The gate voltages were selected to induce a hole gas of varying density in the bP FET. The magnetoresistance exhibits a weak localization peak at low field *B*<1T, a smooth positive magnetoresistance background, and Shubnikov-de Haas (SdH) oscillations at fields exceeding ∼15 T. The SdH oscillations were analysed by fitting the resistance *R*_2P_(*B*) to a parabolic function, as shown in Fig. 3a, to subtract the smooth magnetoresistance background. The resultant oscillating magnetoresistance component Δ*R*_2P_ is plotted versus 1/*B* in Fig. 3b. The low carrier density and high magnetic field regime of our experiments lead to the approximation Δ*R*_2P_ ∝ Δ*R*_xx_ ∝ Δ*σ*_xx_, from which a Lifshitz–Kosevich (LK) form of SdH oscillations follows, Δ*R*_2P_=*R*_D_(*B*)cos[2*π*(*B*_F_/*B*+1/2+*β*)] where *B*_F_ is the magnetic frequency, *β* is the normalized Berry phase and *R*_D_(*B*) is the damping factor arising from hole scattering and finite temperature^17^. We model the damping factor of SdH oscillations with *R*_D_(*B*)=*R*_S_ × Λ/sinh(Λ) where *R*_S_=exp(−2*π*^2^m*k_B_*T*_D_/*ℏeB*) is the Dingle factor and Λ=2*π*^2^m*k_B_*T*/*ℏeB* parameterizes thermal damping. The Dingle temperature *T*_D_ is related to the hole scattering time *τ*=ℏ/2*πk*_B_*T*_D_ and m* is the effective mass for in-plane cyclotron motion. An example of a best fit of the SdH oscillations to the LK form is indicated with a dashed line in Fig. 3b.

The longitudinal resistance *R*_XX_ of the 43±2 nm thick Hall bar bP FET, measured by an ac lock-in technique with a 1 *μ*A bias current, also exhibits SdH oscillations versus magnetic field as shown in Fig. 3c at different gate voltages *V*_g_. The SdH oscillations were analysed by fitting the resistance *R*_XX_(*B*) to a parabolic function to subtract the smooth magnetoresistance background. The resultant oscillating magnetoresistance component Δ*R*_XX_ is plotted versus 1/*B* at *V*_g_ =−100 V versus sample temperature *T* in Fig. 3d. The SdH oscillation amplitude is suppressed as temperature increases. Figure 3e shows the SdH oscillation peaks in Δ*R*_XX_ at magnetic fields *B* =32 T and *B*=26 T versus temperature *T* at *V*_g_=−100 V, along with a best fit to *R*_0_ × Λ/sinh(Λ). The best fit yields an effective mass m*=0.36±0.03*m*_0_ for holes. By comparison, the in-plane effective mass determined by cyclotron resonance experiments of bulk bP is 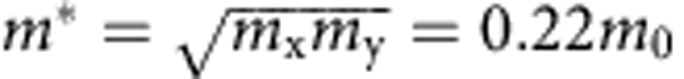 (ref. 12). Temperature-dependent measurements of SdH oscillations of quantum confined holes in bP FETs performed independently by Li *et al*.^13^ yield an effective mass m*=0.34*m*_0_. In agreement with the work of Li *et al*., we observe an enhancement in hole effective mass over that in bulk bP.

At the lowest temperature *T*=300 mK, the damping of SdH oscillations is disorder limited. The LK best fit to SdH oscillations at an applied gate voltage *V*_g_=−100 V corresponds to a Dingle temperature of *T*_D_=10 K for our 47±1 nm thick two-terminal bP FET and *T*_D_=20 K for our 43±2 nm thick Hall bar bP FET. The corresponding hole scattering times *τ* =*ℏ*/2*πk*_B_*T*_D_=60–120 fs and magneto-resistive mobilities *μ*_MR_=*eτ*/m*=300–600 cm^2^ V^−1^ s^−1^. As expected, the magneto-resistive mobilities are less than the observed field effect mobilities because of the shorter scattering time that broadens Landau levels versus the transport scattering time.

### Landau fan diagram

**Figure 4:**
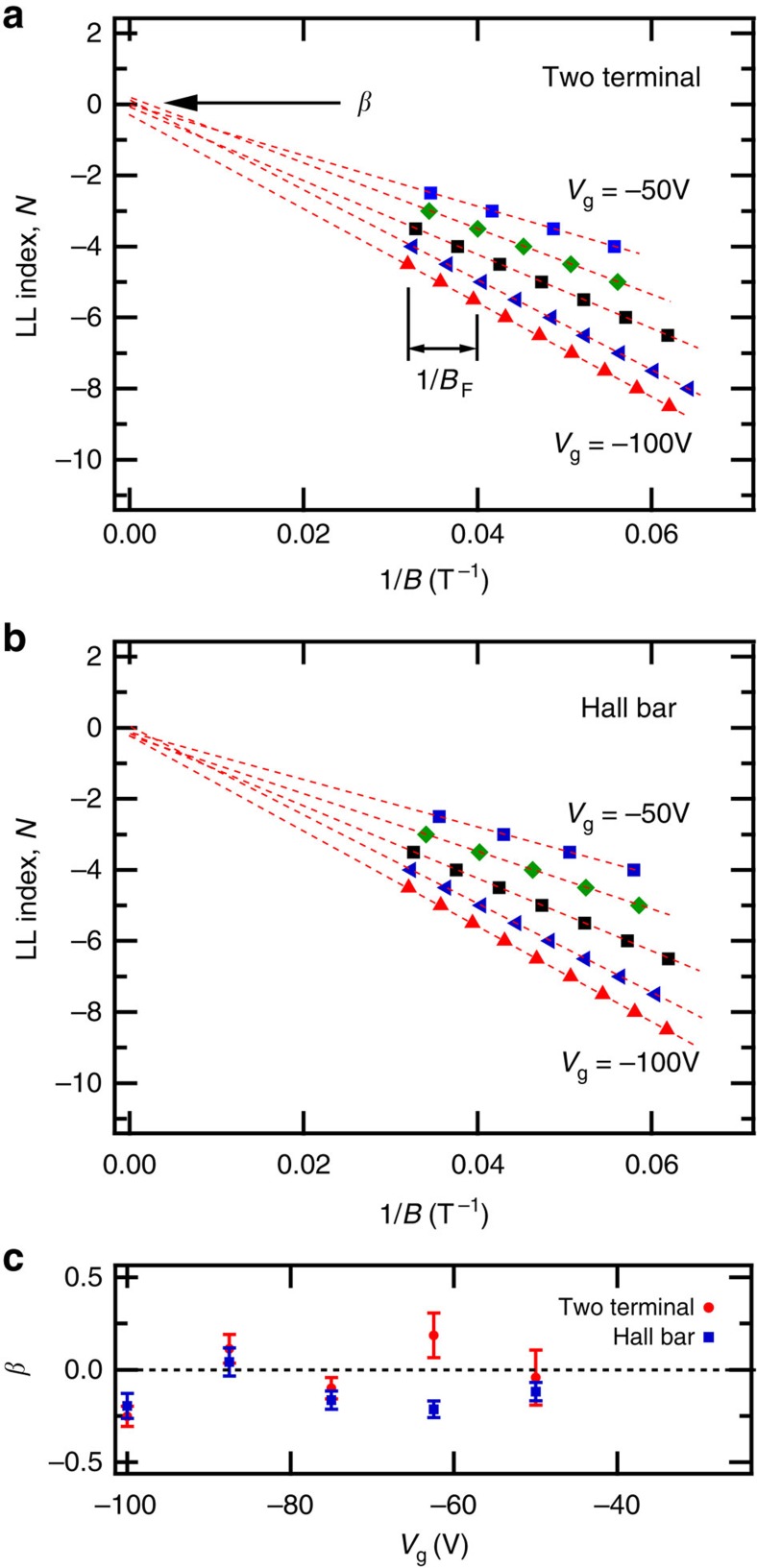
Landau fan diagram analysis. (**a**,**b**) The Landau fan diagram of LL index *N* versus 1/*B* at different gate voltages extracted from Lifshitz-Kosevich analysis of the SdH oscillations of Δ*R* for a two-terminal bP FET and Hall bar bP FET, respectively. Linear best-fit to LL index *N* versus 1/*B* are indicated with dashed lines for each gate voltage *V*_g_=−50 V through −100 V. The SdH frequency *B*_F_ is extracted from the slope of each line. The Berry phase *β* is determined from the LL index intercept of each line at *B*=0, as highlighted by the arrow. (**c**) The Berry phase *β* versus gate voltage *V*_g_ for both devices with wost-case error bars determined from the maximum deviation in the 1/B corresponding to the extrema of the measured SdH oscillations and the extrema of the Lifshitz–Kosevich fit to SdH oscillations. Our results are consistent with *β*=0 for Schrödinger fermions.

The Landau levels (LLs) at the origin of SdH oscillations can be further analysed with a Landau fan diagram of LL index *N* versus 1/*B*, illustrated in Fig. 4a,b for the two-terminal bP FET and Hall bar bP FET, respectively. The LL index *N* corresponds to the *N*^th^ minimum in Δ*R* versus 1/*B*. The half integer index *N*+1/2 corresponds to the *N*^th^ maximum in Δ*R*. The LK fits to the SdH oscillations at each gate voltage were used to determine the minima and maxima presented in the fan diagrams of Fig. 4a,b. Notably, our experiments closely approach the quantum limit, with an LL index as small as *N*+1/2=2.5 being observed. At each gate voltage, the magnetic frequency *B*_F_ determined by the LK fit corresponds to the slope of the Landau fan diagram *B*_F_=*δN*/*δ*(1/*B*). The Berry phase *β* corresponds to the LL index intercept at 1/*B* =0 in the Landau fan diagram. The Berry phase determined at each gate voltage for two samples is summarized in Fig. 4c, and is consistent with a trivial phase *β*=0. Our results are consistent with the expectation that holes in bP are Schrödinger fermions devoid of pseudo-spin, as there is a single hole valley in bP at the *Z*-point of the first Brillouin zone^2^, and are in agreement with independent measurements of SdH oscillations by Li *et al*.^13^

## Discussion

Information about the hole gas in the bP FET can be determined by comparison of the free-charge density per unit area versus the charge density induced by field effect *n*_FE_=*CV*_g_/*e* where *C* =11.5 nF cm^−2^ is the gate capacitance per unit area of 300 nm of SiO_2_. In the case of a 2D hole gas occupying a single valley at Z with unbroken spin degeneracy, the free-charge density is *n*_free_=2*B*_F_ × *e*/*h*. Spin degeneracy appears unbroken through out the SdH oscillations of Fig. 3. Disorder in our samples, characterized by a field effect hole mobility of up to 900 cm^2^ V^−1^ s^−1^ and a Dingle temperature as low as 10 K, suppresses the observation of spin split LLs. Recently, field effect hole mobilities reaching 3,900 cm^2^ V^−1^ s^−1^ and field effect electron mobilities reaching 1,600 cm^2^ V^−1^ s^−1^ have been observed by Li *et al*.^13^, enabling the direct observation of spin-split LLs in SdH oscillations for both electrons and holes.

**Figure 5:**
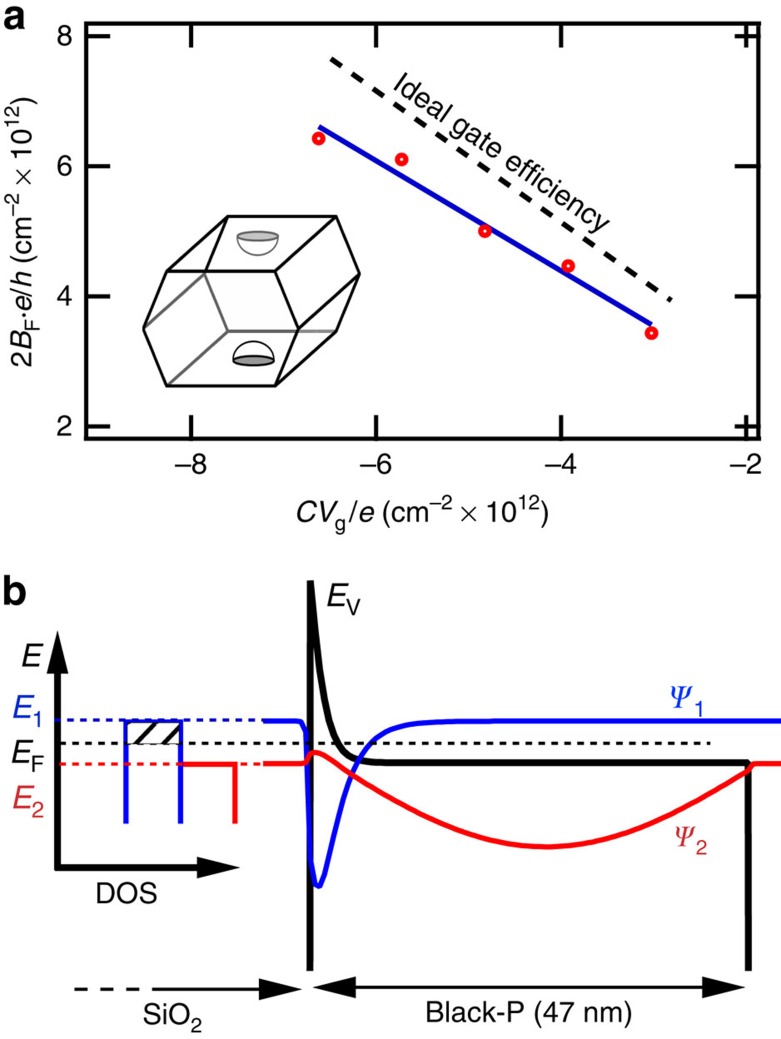
Free-carrier density analysis. (**a**) The free-carrier density 2*B*_F_ × *e*/*h* versus the charge density induced by field effect *CV*_g_/*e* (red circles). A single, spin degenerate hole valley at *Z*, illustrated in the inset, is assumed. A linear fit (blue) corresponds to a 78% gate efficiency. The error in carrier concentration was determined from the standard deviation in the Lifshitz–Kosevich fit for *B_F_*. The error bars are smaller than the symbol size. (**b**) Self-consistent Schrödinger-Poisson calculation of the ground state wavefunction Ψ_1_ (*E*_1_−*E*_F_=13 meV) and first excited state wavefunction Ψ_2_ (*E*_F_−*E*_2_=15 meV) for holes, and the corresponding valence band edge *E*_V_, at a gate bias of *V*_g_=−50 V. A single 2D sub-band is occupied.

The free charge versus gated charge of the two-terminal 47 nm thick bP FET device is plotted in Fig. 5a, along with a linear best fit that corresponds to a 78% gate efficiency. The linearity and proximity to ideal gate behaviour is consistent with the presence of a 2D hole gas. In contrast, the carrier density versus magnetic frequency for a 3D hole gas is given by 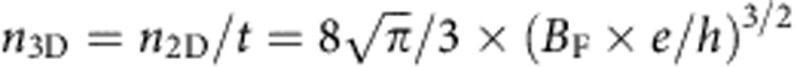 where *t* is the effective thickness of the hole gas^17^,^18^. The measured free-carrier density versus charge density induced by field effect does not agree with a 3D model unless a hole gas thickness of *t* ≈3 nm is assumed, in which case 2D quantum confinement effects must be taken into account. In other words, the observed magnetic frequency variation versus gate voltage indicates the presence of a 2D hole gas rather than a 3D hole gas. Recent magnetotransport measurements of bP FETs in variably tilted magnetic fields by Li *et al*.^13^ are in agreement with our observation of a 2D hole gas.

We estimated the 2D quantum confinement of holes that accumulate at the bP surface under the influence of back-gate potential with a self-consistent Schrödinger-Poisson calculation using an effective mass band theory. Estimates of the occupied and un-occupied 2D sub-band wavefunctions, valence band edge and density of states are illustrated in Fig. 5b at a gate bias of *V*_g_=−50 V. The hole gas in the wide bP quantum well is similar to the 2D accumulation (or inversion) layer induced in Si FETs at an Si / SiO_2_ interface. The strong electric field, of order ∼0.1 V nm^−1^, applied to induce a hole gas within the bP results in a 2D sub-band with a wavefunction Ψ_1_ that is tightly confined to an r.m.s. width of 2.7 nm (∼5–6 atomic layers). At *V*_g_ =−50 V, Schrödinger-Poisson calculation places the Fermi level *E*_F_ 13 meV below the ground state sub-band edge *E*_1_, but the first excited sub-band *E*_2_ lies 15 meV below the Fermi level and is thus energetically inaccessible to holes at *T*=0.3 K. The bP/oxide interface is expected to be rich in charge traps, and is consistent with the modest mobilities and deviation from ideal gate efficiency observed for the 2D hole gas confined to the bP/oxide interface in our work. However, the saturation of in-plane chemical bonding within bP likely leads to a low density of dangling bonds that would otherwise lead to Fermi level pinning and suppression of the electric field effect.

We have thus shown that two distinct aspects of 2D physics can co-exist in one material system. On the one hand, the 2D nature of covalent bonding in bP is favourable for mechanical exfoliation of thin layers and results in a low dangling bond density at naked bP surfaces. On the other hand, the field effect can be used to induce a charge accumulation layer in a single 2D electronic sub-band as in conventional semiconductor heterostructures and quantum wells. 2D electronic behaviour can be assessed in multi-layer devices, and this degree of freedom is anticipated to be useful in the future development of 2D electronics, including van der Waals heterostructures that combine multiple layered materials together. In the case of layered semiconductor materials whose stability decreases as the few-layer limit is approached such as bP, multi-layer stacks can be used while retaining 2D electronic behaviour.

## Methods

### Device fabrication

The source material for bP preparation were 99.998% purity bP crystals from Smart Elements (Vienna, Austria). Mechanical exfoliation of bP layers was performed using a PDMS stamp technique within a nitrogen glove box environment to minimize exposure to water and oxygen^6^. A multi-step process was used, wherein bP was first exfoliated directly to a strip of adhesive tape. The bP was then repeatedly exfoliated 15 times with the strip of adhesive tape. A flat PDMS stamp was then used to exfoliate bP from the adhesive tape five times, and finally the flat PDMS stamp was used to deliver bP to target sample substrates. The sample substrates were degenerately doped Si wafers, with 300 nm of chlorinated dry thermal oxide. The substrates were pre-patterned with metal alignment marks and were annealed at 150 °C for 15 min to desorb water before bP exfoliation. Optical reflection microscopy with red light (using a 580-nm-long pass filter) was performed to identify thin bP flakes while minimizing the effects of photo-oxidation. Conventional electron beam lithography and metal deposition were used to define Ti/Au (5 nm/ 80 nm) contacts on bP flakes, with care taken to minimize simultaneous exposure to water, oxygen and visible light. Once fabricated, the bP FETs were encapsulated in a glove box environment by spin-coating 300 nm of copolymer (methyl methacrylate) and 200 nm of polymer (polymethyl methacrylate) followed by an annealing step at 170 °C for 15 min.

### Quasi-DC characterization

**Figure 6:**
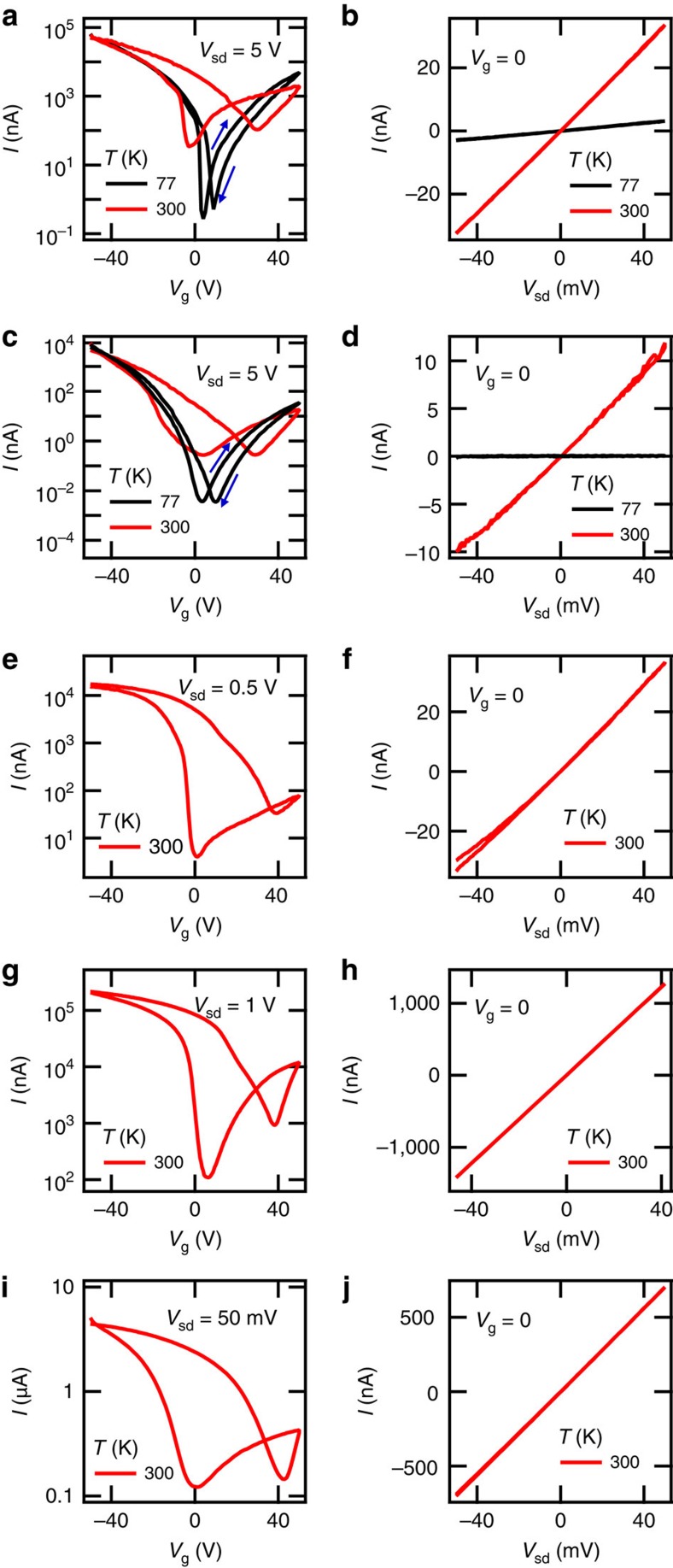
Quasi-dc FET characterization. (**a**,**c**,**e**,**g**,**i**) The source-drain current *I* of two-terminal FETs of 11.5±1 nm, 6±1 nm, 12.5±1 nm, 47±1 nm bP thickness and Hall bar FET of 43±2 nm bP thickness, respectively, at fixed source-drain bias *V*_sd_ versus gate voltage *V*_g_. room temperature and 77 K measurements are presented for devices of thickness 11.5±1 nm and 6±1 nm, showing a decrease in hysteresis and increase in on/off ratio at 77 K. (**b**,**d**,**f**,**h**,**j**) The source-drain current *I* of devices of 11.5±1 nm, 6±1 nm, 12.5±1 nm and 47±1 nm thickness, respectively, with a gate bias of *V*_g_=0 V versus source-drain bias *V*_sd_. Ohmic behaviour is observed.

Initial charge transport experiments on the bP FETS were performed under quasi-dc bias in a vacuum probe station with a semiconductor parameter analyser. The characteristics of the representative bP FETs are displayed in Fig. 6. The source-drain current *I* was measured at fixed source-drain bias *V*_sd_ versus gate voltage *V*_g_ swept in both directions. Most devices exhibited ambipolar conduction and an on/off current ratio that increased as temperature was decreased from 300 to 77 K. As seen in Fig. 6, hysteresis was also observed to rapidly decrease as temperature decreased. Gate leakage current was simultaneously monitored in all experiments and never exceeded 10% of the minimum source-drain current. The source-drain current versus bias voltage *V*_sd_ was also measured for all devices as shown in Fig. 6b.

### AC characterization

**Figure 7:**
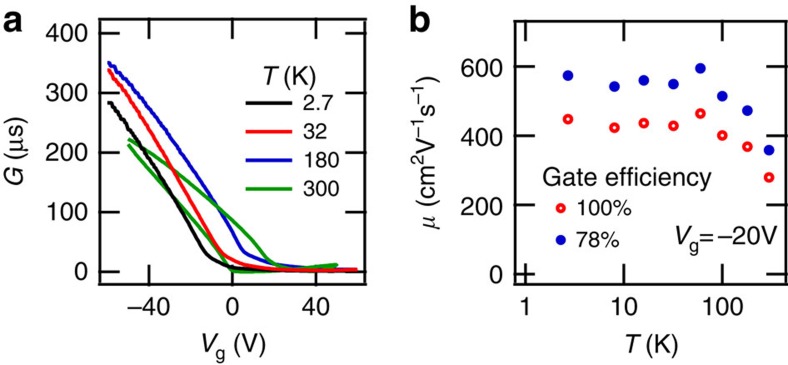
Field effect mobility. (**a**) The ac conductance *G* versus gate voltage *V*_g_ at several different temperatures *T* for a two-terminal device of 47±1 nm bP thickness. Hysteresis observed at room temperature is suppressed at low temperature. (**b**) Field-effect mobility *μ*_FE_ at fixed gate voltage *V*_g_ =−20 V versus temperature *T* assuming 100% gate efficiency and a gate efficiency of 78% as determined from SdH frequency analysis versus gate voltage. The error in mobility was determined by the noise in the measured conductance. The error bars are smaller than the symbol size.

Following quasi-dc characterization, devices were selected for characterization over a wider temperature range. Samples were mounted on fibre glass chip-carriers and electrical contact made by wire bonding. A variable temperature insert in a helium cryostat was used to measure source-drain resistance down to temperatures of 2.5 K using standard ac lock-in measurement with a 10 mV voltage bias. The field effect mobility *μ*_FE_=(*L*/*W*) × ∂*G*/∂(*CV*_g_) was determined from the measured ac conductance *G* of a two-terminal device plotted in Fig. 7a. The mobility assuming 100% gate efficiency and 78% gate efficiency *C* → 0.78 × *C* as determined from LK analysis are plotted in Fig. 7b versus temperature. Mobility decreases with temperature for *T* >100 K, and is independent of temperature for *T* <100 K, in agreement with other observations of bp FETs^13^,^14^,^15^. Magnetotransport measurements were conducted in a helium-3 cryostat in a resistive magnet cell at the National High Magnetic Field Laboratory (Tallahassee, Florida). During all magnetotransport measurements, the samples were immersed in a helium-3 bath at 300 mK. Source-drain resistance of a two-terminal bP FET and longitudinal resistance of a Hall bar bP FET were measured using standard ac lock-in techniques. All data are available upon request to T.S.

### Raman spectroscopy

**Figure 8:**
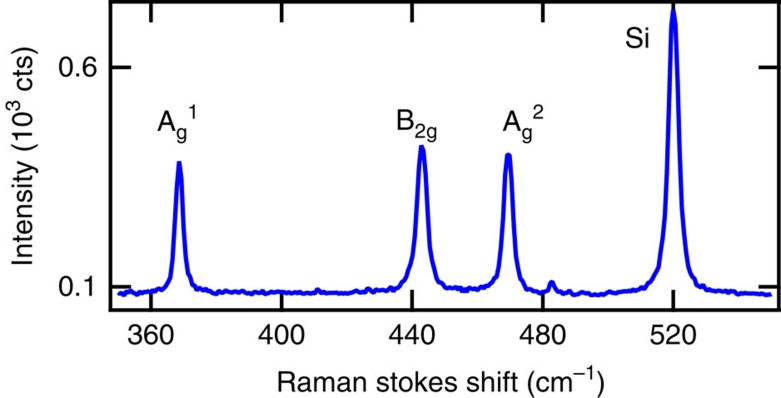
Raman spectroscopy. Raman Stokes shift of encapsulated bP FET with 6±1 nm bP thickness measured with a 532 nm laser pump. A silicon substrate peak is observed as well as the 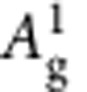, *B*_2g_ and 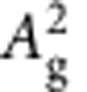 Raman modes of bP.

Raman spectroscopy was performed once all electron transport experiments were completed to minimize the effect of photo-oxidation on the electronic quality of the bp FETs. Raman spectroscopy was performed with the sample in a vacuum cell using a custom built instrument with a laser pump at 532 nm and a numerical aperture of NA=0.55. The pump fluence is estimated to be 20 kW cm^−2^. The resolution of the Raman spectrometer is ±0.2 cm^−1^. A representative Raman Stokes spectrum of an 6±1 nm bp FET is shown in Fig. 8. The strong peak at 520 cm^−1^ originates from the Si substrate^19^. The three peaks observed at 368.7, 442.8 and 469.2 cm^−1^ correspond to the 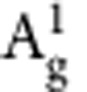, B_2g_ and 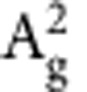 Raman modes of bP as reported in studies of bulk, single-crystal bP^20^.

### Atomic force microscopy

**Figure 9:**
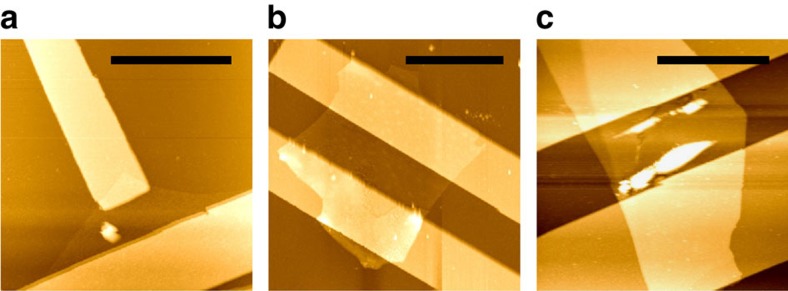
AFM images. (**a**,**b**,**c**) AFM images of unencapsulated bP FET devices with respective thicknesses of 6±1 nm, 12.5±1 nm and 47±1 nm. The scale bars are 4, 8 and 8 μm, respectively.

The PMMA/MMA superstrate layers were removed from bP FETs by immersion in acetone in a glove box environment. AFM was performed within the same glove box with a ThermoMicroscopes Auto Probe CP to determine the thickness of the bP layers. AFM images were acquired in intermittent imaging mode with 85% damping with Al-coated Si cantilever probes (tip radius <10 nm, spring constant 25–75 N m^−1^). AFM images are shown in Fig. 9, from which thicknesses of 6±1, 12.5±1 and 47±1 nm were determined, respectively.

### Schrödinger-poisson analysis

The nature of the 2D hole gas accumulating at the bP surface by action of the gate potential was modelled with self-consistent Schrödinger-Poisson calculations. The calculations were performed in one dimension by iterative solution of an effective mass Schrödinger equation and Poisson's equation for the mean-field electrostatic potential of the hole density. Calculations were performed with a bP effective hole mass of 0.28*m*_0_, a bP dielectric constant 6.1*ɛ*_0_ (ref. 21), where *ɛ*_0_ is the permittivity of free space, and 3 eV potential barriers at the bP surface as a model for a hard potential barrier into the oxide substrate or polymer superstrate. Fermi-Dirac statistics at 3 K were used for hole population, and the field effect was modelled by including an applied electric field.

## Additional information

**How to cite this article:** Tayari, V. *et al*. Two-dimensional magnetotransport in a black phosphorus naked quantum well. *Nat. Commun.* 6:7702 doi: 10.1038/ncomms8702 (2015).
